# Association between residential greenness and cognitive function: analysis of the Chinese Longitudinal Healthy Longevity Survey

**DOI:** 10.1136/bmjnph-2019-000030

**Published:** 2019-08-26

**Authors:** Anna Zhu, Chenkai Wu, Lijing L Yan, Chih-Da Wu, Chen Bai, Xiaoming Shi, Yi Zeng, John S Ji

**Affiliations:** 1 Environmental Research Center, Duke Kunshan University, Kunshan, China; 2 Global Health Research Center, Duke Kunshan University, Kunshan, China; 3 Department of Geomatics, National Cheng Kung University, Tainan, Taiwan; 4 Department of Social Security, Renmin University of China, Beijing, China; 5 National Institute of Environmental Health, Chinese Center for Disease Control and Prevention, Beijing, China; 6 Center for the Study of Aging and Human Development, Duke University School of Medicine, Durham, North Carolina, USA; 7 Center for Healthy Aging and Development Studies, and Raissun Institute for Advanced Studies, Peking University, Beijing, China; 8 Nicholas School of the Environment, Duke University, Durham, United States

**Keywords:** residential greenness, Normalised Difference Vegetation Index, cognitive function, Mini-Mental State Examination (MMSE), China, ageing, environmental epidemiology

## Abstract

**Introduction:**

Proximity to vegetated green space has been linked to better physical and mental health. However, the relationship between residential greenness and cognitive function and its decline among older adults is not clear in large cohort studies.

**Methods:**

Our study used the 2000, 2002, 2005, 2008 and 2011 wave of the Chinese Longitudinal Healthy Longevity Survey. We calculated the Normalised Difference Vegetation Index (NDVI) using a 500 m radius around participants’ residential addresses. Mini-Mental State Examination (MMSE) was applied to measure cognitive function. Our study included the cross-sectional analysis using the linear regression, and logistical regression, and also the longitudinal analysis using the linear mixed effects regression, and mixed effects logistic regression. Our study also conducted a sensitivity analysis using the survey-weighted regression. Additionally, our study participants were categorised into those living in areas of positive and negative changes in NDVI in relation to MMSE. All regression models were adjusted for a range of covariates.

**Results:**

Among 38 327 participants at baseline, the mean MMSE score was 21. Annual average NDVI ranged from −0.11 to 0.76. In the cross-sectional analysis, each 0.1-unit increase in NDVI was associated with a 0.23-point increase in MMSE score (95% CI 0.16 to 0.29) in the linear regression, and an OR of 0.94 (95% CI 0.92 to 0.96) of having cognition impairment in the logistic regression. In the second analysis, looking at changes in NDVI and MMSE score, compared with the participants living in areas with an increase in NDVI, those living in areas with a decrease in greenness had an OR of 1.25 (95% CI 1.18 to 1.34) of a decrease in MMSE, and an OR of 0.90 (95% CI 0.84 to 0.96) of an increase in MMSE. In the longitudinal analysis, we found a significantly weak association (coefficient 0.069, 95% CI 0.0048 to 0.13) in the linear mixed effects regression, but not in the mixed effects logistic regression.

**Conclusion:**

We found evidence of an association between higher residential greenness and better cognitive function among older adults. Our finding provides insight into neurodegeneration and has implications for preventing dementia and Alzheimer’s disease in China.

**What this paper adds**

- There was a protective association between residential greenness and cognitive function among older adults.
- Reduced residential greenness was associated with a higher odds of cognitive decline.
- Increasing green space may reduce cognitive decline, and subsequently disease burden of dementia in China.

## Introduction

Cognitive function declines with age, and is a precursor for dementia and Alzheimer’s disease (AD).^1 2^ As life expectancies increase globally, loss of cognition is poised to increase its share on the burden of diseases. In several independent cohorts, living in areas with higher greenness exposure was linked to better physical and mental health.^3–5^ The natural environment may affect cognitive function, supported by the attention restoration theory and the stress reduction theory. Attention restoration theory posits that exposure to nature stimulates usage of the non-directed concentration not normally used, which allows the directed attention to replenish and restore.^6^ Stress reduction theory states that exposure to nature helps to mitigate self-reported stress and promote a positive mood.^6 7^

There is evidence of an association between greenness and cognitive function in population health studies, but the relationship is not clear. Protective associations were reported in two studies of 884 adults aged 65 years and older in the USA,^8^ and 1091 adults aged 70 years and older in Scotland.^9^ However, no association was found in 949 adults aged 50 years and older in Chicago, USA,^10^ and 6658 adults aged 40–69 years in Quebec, Canada.^11^ In contrast, higher levels of residential greenness were associated with higher odds of cognitive impairment in 2424 participants aged 65 years and older in the UK.^12^

In the present study, we hypothesised that higher levels of residential greenness were associated with better cognitive function among Chinese older adults. China has the largest number of older adults aged 65 years and older (about 148 million in 2017),^13^ and the largest number of older adults with AD and dementia (14.9 million in 2010) in the world.^14^ Evidence on whether the built environment can affect cognitive function has important public health implications.

## Methods

### Study population

We used five waves (2000, 2002, 2005, 2008 and 2011) from the Chinese Longitudinal Healthy Longevity Survey (CLHLS). Launched in 1998, the CLHLS aimed to study determinants of healthy longevity among Chinese older adults. The CLHLS has a nationally representative sample of older adults aged 65 years and older, with participants from 22 out of 31 provinces in China. The CLHLS has a higher proportion (75%) of older adults aged 80 years and older due to its sampling design. The CLHLS is a multistage, stratified cluster survey conducted in 631 randomly selected cities and counties where the Han Chinese are the largest majority. The sample sites represent about 85% of the Chinese population. A more detailed description of the sampling design can be found elsewhere.^15^

The CLHLS collected extensive data, including demographic characteristics, socioeconomic status (SES), lifestyle, physical capacity, cognitive function, psychological status and mortality.^14^ We had 39 225 participants in five pooled waves. We excluded participants who were missing residential addresses (n=348), younger than 65 years at baseline (n=545) and missing all Normalised Difference Vegetation Index (NDVI) values (n=5). Our study sample had 38 327 participants in the baseline analysis. A total of 19 726 participants had follow-up surveys, and 18 601 participants had only one survey. A total of 12 522 participants died, and 6079 participants were lost to follow-up. The characteristics of those with a follow-up survey and those without a follow-up survey can be seen in online supplementary table 1.

10.1136/bmjnph-2019-000030.supp1 Supplementary data

### Greenness assessment

We used the NDVI to measure residential greenness. NDVI is a satellite image-based vegetation index obtained from the Moderate Resolution Imaging Spectroradiometer (MODIS) in the National Aeronautics and Space Administration’s Terra satellite.^16 17^ The measurement of NDVI is based on chlorophyll in plants, which absorbs visible light for photosynthesis, while leaves reflect near-infrared light. NDVI is equals to the ratio of the difference between the near-infrared region and red visible reflectance to the sum of these two measures. NDVI ranges from −1.0 to 1.0, with larger values indicating higher levels of vegetative density. Negative values often refer to blue space or water; values of 0.1 and below reflect barren areas of rock, sand or snow; values of 0.2–0.4 represent shrub and grassland; while higher values indicate temperate and tropical rainforests.^18 19^

The CLHLS collected residential addresses from a large sample size of participants, who were from geographically diverse regions of China. We calculated NDVI values in the 500 m radius based on the longitude and latitude of each residential address. Evidence shows that 0.25 miles (400 m), which is about a 5 min walking distance, is not a necessary driving distance. A number of studies used the 500 m radius as the acceptable walking distance around the residence. We think that the 500 m radius is optimal to reflect residential greenness.^20^ MODIS has a temporal resolution of 16 days. We calculated two NDVI values for each month. We assessed NDVI values in January, April, July and October from 2000 to 2014 to reflect greenness of four seasons. We calculated annual average NDVI for the year of study entry year to reflect residential greenness at baseline; of the last survey among participants with follow-up surveys, to reflect residential greenness in a later stage. Changes in NDVI was the difference in annual average NDVI values measured between at the last survey and the baseline. Additionally, we calculated 0.1-unit and quartiles of NDVI values.

### Cognitive function assessment

We assessed cognitive function using the adapted Chinese version of the Mini-Mental State Examination (MMSE) with 24 self-reported questions.^15^ MMSE assesses cognitive function in five dimensions: orientation, registration, attention and calculation, recall and language.^15 21^ Each question was scored 0 (wrong or unable to answer) or 1 (correct).^22 23^ We converted the 24-item MMSE to a scale from 0 to 30 for consistency with other studies.^21 23 24^ Higher scores indicated better cognitive function.^24^ We dichotomised MMSE scores, and defined scores of ≥24 as normal cognition (as the reference group) and <24 as cognitive impairment. We used three outcome measures: baseline MMSE, to reflect cognitive function at study entry; final MMSE, to reflect cognitive function at last survey; and changes in MMSE throughout the follow-up period. Changes in MMSE was the difference in MMSE scores measured between at the last survey and the baseline, which were categorised as no change, a decrease or an increase.

### Covariates

We assessed a range of baseline characteristics including age, gender, ethnicity, marital status, geographic region, urban/rural residence, education, occupation, financial support, social and leisure activity, smoking status, alcohol consumption and physical activity. We calculated age using the interview dates, and the birth dates (converted from the lunar calendar when necessary) verified through family members, genealogical records, ID cards and household registration booklets. We dichotomised ethnicity to Han Chinese and ethnic minorities (including Hui, Korean, Manchurian, Mongolian, Yao, Zhuang and others). We coded marital status as a binary variable: married and living with their spouse, or not married at the time of interview (separated, or divorced, or widowed, or never married). Based on the residential addresses of participants, we divided them into seven geographical regions to reflect differences in climate and dietary patterns: Central China (Henan, Hubei and Hunan provinces), Eastern China (Anhui, Fujian, Jiangxi, Jiangsu, Shandong, Shanghai and Zhejiang provinces), Northeastern China (Heilongjiang, Jilin and Liaoning provinces), Northern China (Hebei, Shanxi and Tianjin provinces), Northwestern China (Shaanxi province), Southern China (Guangdong, Guangxi and Hainan provinces) and Southwestern China (Chongqing and Sichuan provinces). We defined residence depending on whether the participants lived in urban or rural areas. We generated a binary variable of education, some formal education (≥1-year schooling) or no formal education, based on years of schooling. We categorised occupation into professional (professional and technical personnel, government and management) and non-professional work (agriculture, fishing, service, industry and housework). We dichotomised financial support to financial independence if the participants had their work and retirement wage, and financial dependence if they relied on other family members. We assessed the social and leisure activity index by seven activities: gardening, personal outdoor activities excluding exercise, raising poultry or pets, reading, playing cards or *Mahjong*, listening to the radio or watching TV and participating in organised social activities.^25^ Each activity was scored 0 (no) or 1 (yes), and the index ranged from 0 to 7. We evaluated smoking status, alcohol consumption and physical activity by the questions of ‘Do you smoke/drink/exercise or not at present’.

### Statistical analysis

Our study used linear regression, logistic regression, linear mixed effects regression and mixed effects logistic regression models to assess the associations between residential greenness and cognitive function, adjusted for age, gender, ethnicity, marital status, geographic region, urban/rural residence, education, occupation, financial support, social and leisure activity, smoking status, alcohol consumption and physical activity. First, a cross-sectional analysis was conducted using a linear regression of baseline year annual average NDVI and baseline MMSE score, and a logistic regression of baseline year annual average NDVI and cognitive impairment. Second, the participants were categorised into those living in areas of positive and negative changes in NDVI in relation to MMSE. The participants with no change or an increase in MMSE, and no change or a decrease in MMSE were defined as the reference group in two separate models. Third, a longitudinal analysis was performed using linear mixed effects regression, and mixed effects logistic regression models of baseline year annual average NDVI and MMSE among participants with a follow-up survey. The longitudinal analysis was additionally adjusted for the time to reflect the number of years for each follow-up survey since entering the cohort. Fourth, because the oldest-old were oversampled in the CLHLS, we conducted a sensitivity analysis on the relationship between residential greenness and cognitive function, incorporating sampling weights. The participants with normal cognition (MMSE≥24) were considered as the reference group. We calculated coefficients, ORs and 95% CIs to estimate magnitude and odds of cognitive impairment under different levels of residential greenness. All statistical analysis was conducted using STATA V.14.0.

### Patient and public involvement statement

CLHLS was conducted among older adults but not the specific patients. According to the requirement of ethic committee, informed consent was needed no matter whether the participants were patients or not. We indicated informed content rather than patient consent.

## Results

Table 1 presents the baseline characteristics of the 38 327 participants. Their mean age was 88 (SD 11.45) years, and 77.75% of them were aged 80 years and older. We had more participants who were females (58.91%) and resided in rural areas (76.32%). The mean and median MMSE score was 21 (SD 9.72) and 25 (IQR 15–29), respectively. Mean annual average NDVI in the 500 m radius was 0.40 (SD 0.15). Compared with people living in less green areas, those living in greener areas tended to be females, from ethnic minorities, unmarried, rural residents, with non-professional work, uneducated, financially dependent, smoking, drinking and physically inactive. Participants with higher MMSE scores were more likely to be younger, males, married and living with their spouse, living in urban areas, with professional work, educated, financially independent, smoking, drinking and physically active, than were those with lower MMSE scores.

**Table 1:** Baseline characteristics of participants

Characteristics	Total (n, %)	Annual average NDVI (mean±SD)	MMSE scores (mean±SD)
	38 327	0.40±0.15	21±9.72
Age (years) (mean±SD)	88±11.45	/	/
Age group (years)			
65–79	8528 (22.25)	0.38±0.15	27±4.11
80–89	10 161 (26.51)	0.40±0.14	24±7.14
90–99	10 418 (27.18)	0.40±0.15	19±9.80
≥100	9220 (24.06)	0.40±0.15	13±10.31
Gender			
Males	15 747 (41.09)	0.39±0.15	24±8.37
Females	22 580 (58.91)	0.40±0.15	19±10.04
Ethnicity			
Han Chinese	36 156 (94.34)	0.39±0.15	21±9.76
Ethnic minorities	2171 (5.66)	0.46±0.13	21±9.02
Marital status			
Married and living with their spouse	10 287 (26.84)	0.38±0.15	26±6.52
Not married	28 040 (73.16)	0.40±0.15	19±10.08
Residence			
Urban area	9076 (23.68)	0.24±0.13	21±9.87
Rural area	29 251 (76.32)	0.45±0.12	20±9.66
Occupation			
Professional work	2659 (6.94)	0.31±0.16	26±6.92
Non-professional work	35 668 (93.06)	0.40±0.15	20±9.77
Education			
Formal education	13 347 (34.82)	0.37±0.16	25±7.68
No formal education	24 980 (65.18)	0.41±0.14	18±9.96
Financial support			
Financial independence	9142 (23.85)	0.33±0.16	26±6.86
Financial dependence	29 185 (76.15)	0.42±0.14	19±9.96
Social and leisure activity index (mean±SD)	2.07±1.55	/	/
Smoking status			
Yes	6804 (17.75)	0.41±0.14	24±8.16
No	31 523 (82.25)	0.40±0.15	20±9.91
Alcohol consumption			
Yes	7383 (19.26)	0.41±0.14	23±8.87
No	30 944 (80.74)	0.39±0.15	20±9.85
Physical activity			
Yes	10 923 (28.50)	0.36±0.15	24±7.38
No	27 404 (71.50)	0.41±0.14	19±10.11
Geographic region			
Central China	5580 (14.56)	0.44±0.12	20±9.84
Eastern China	15 491 (40.42)	0.40±0.16	21±9.60
Northeastern China	2922 (7.62)	0.28±0.11	20±10.58
Northern China	1922 (5.01)	0.26±0.11	21±9.81
Northwestern China	512 (1.34)	0.37±0.14	21±8.53
Southern China	6931 (18.08)	0.44±0.13	21±9.21
Southwestern China	4969 (12.96)	0.40±0.13	20±10.06

MMSE, Mini-Mental State Examination; NDVI, Normalised Difference Vegetation Index.

Table 2 presents the association between residential greenness and cognitive function. At baseline, in the adjusted linear regression, each 0.1-unit increase in NDVI was associated with a 0.23-point higher MMSE score (95% CI 0.16 to 0.29). In the adjusted logistic regression, per 0.1-unit increase in NDVI was associated with an OR of 0.94 (95% CI 0.92 to 0.96) of cognitive impairment. Compared with the participants living in the lowest quartile of residential greenness, those in the highest quartile had a 25% (95% CI 0.69 to 0.82) lower odds of cognitive impairment. In the adjusted linear mixed effects regression, we observed a weak association between NDVI and MMSE scores among the participants with follow-up survey (coefficient 0.069, 95% CI 0.0048 to 0.13). We did not find a similar association in the adjusted mixed effects logistic regression (OR 0.99, 95% CI 0.97 to 1.01). We also did not find appreciable differences when incorporating sampling weights in the sensitivity analysis (see online supplementary table 2). Furthermore, the subgroup analysis did not show consistent findings on effect modification by gender, and urban/rural residence (table 3), in the cross-sectional analysis, and the longitudinal analysis. In the cross-sectional analysis, residential greenness seemed only to benefit participants who were physically inactive at baseline.

**Table 2:** Association between residential greenness and cognitive function

Exposure metric	Serial cross-sectional analysis (n=38 327)		Longitudinal analysis (n=19 726)	
	Linear regression	Logistic regression	Linear mixed effects regression	Logistic mixed effects regression
	Coefficient for change in MMSE score (95% CI)	OR of cognitive impairment (95% CI)	Coefficient for change in MMSE score (95% CI)	OR of cognitive impairment (95% CI)
0.1-unit of NDVI	0.23 (0.16 to 0.29)	0.94 (0.92 to 0.96)	0.069 (0.0048 to 0.13)	0.99 (0.97 to 1.01)
Quartiles of NDVI				
Quartile 1	Reference	Reference	Reference	Reference
Quartile 2	0.23 (−0.015 to 0.48)	0.99 (0.92 to 1.08)	−0.046 (−0.29 to 0.19)	1.08 (1.00 to 1.18)
Quartile 3	0.24 (−0.027 to 0.50)	0.93 (0.86 to 1.02)	−0.019 (−0.27 to 0.23)	1.04 (0.95 to 1.14)
Quartile 4	0.99 (0.72 to 1.26)	0.75 (0.69 to 0.82)	0.33 (0.068 to 0.58)	0.92 (0.84 to 1.01)

All the regression models were adjusted for age, gender, ethnicity, marital status, urban/rural residence, education, occupation, financial support, social and leisure activity, smoking status, alcohol consumption and physical activity at baseline. The longitudinal analysis was additionally adjusted for the time to reflect the number of years for each follow-up survey since entering the cohort.

MMSE, Mini-Mental State Examination; NDVI, Normalised Difference Vegetation Index.

**Table 3:** Subgroup analysis for residential greenness and cognitive function

Covariates	Serial cross-sectional analysis (n=38 327)		Longitudinal analysis (n=19 726)	
	Linear regression	Logistic regression	Linear mixed effects regression	Mixed effects logistic regression
	Coefficient for change of MMSE score (95% CI)	OR of cognitive impairment (95% CI)	Coefficient for change of MMSE score (95% CI)	OR of cognitive impairment (95% CI)
**Stratified by gender**				
Males (n=15 747)	0.20 (0.11 to 0.30)	0.93 (0.90 to 0.96)	0.083 (−0.0042 to 0.17)	0.98 (0.95 to 1.02)
Females (n=22 580)	0.24 (0.15 to 0.33)	0.94 (0.92 to 0.97)	0.060 (−0.032 to 0.15)	0.99 (0.96 to 1.02)
P value for interaction	0.31	0.003	0.051	0.044
**Stratified by residence**				
Urban area (n=9076)	0.36 (0.24 to 0.49)	0.92 (0.89 to 0.96)	0.050 (−0.025 to 0.12)	0.99 (0.94 to 1.04)
Rural area (n=29 251)	0.16 (0.081 to 0.24)	0.95 (0.93 to 0.97)	0.11 (−0.023 to 0.24)	0.99 (0.96 to 1.02)
P value for interaction	0.007	0.168	0.333	0.902
**Stratified by physical activity**				
Exercise (n=10 923)	0.084 (−0.013 to 0.18)	0.97 (0.94 to 1.01)	0.027 (−0.069 to 0.12)	1.02 (0.98 to 1.06)
Do not exercise (n=27 404)	0.24 (0.16 to 0.33)	0.93 (0.90 to 0.95)	0.069 (−0.015 to 0.15)	0.97 (0.95 to 1.00)
P value for interaction	0.025	<0.001	0.136	0.009

All the regression models on 0.1-unit NDVI and cognitive function were adjusted for age, gender, ethnicity, marital status, urban/rural residence, education, occupation, financial support, social and leisure activity, smoking status, alcohol consumption and physical activity at baseline. The longitudinal analysis was additionally adjusted for the time to reflect the number of years for each follow-up survey since entering the cohort.

MMSE, Mini-Mental State Examination; NDVI, Normalised Difference Vegetation Index.

Table 4 reports the relationship between changes in NDVI and changes in MMSE. For changes in NDVI over time, 9729 (49.32%) participants lived in areas that became less green, and 9997 (50.68%) lived in areas that became greener. During the follow-up period, stable, deteriorated and improved cognitive function were observed among 11.32%, 56.99% and 31.69% of the participants, respectively. Compared with the participants living in areas that became greener over time, those living in areas that became less green had a 25% (95% CI 1.18 to 1.34) higher odds of having a declined MMSE scores. In addition, a decline in NDVI was related to a 10% (95% CI 0.84 to 0.96) lower odds of having an increased MMSE score.

**Table 4:** ORs and 95% CI for changes in residential greenness and in cognitive function

Changes in NDVI	Participants	A decrease in MMSE*		An increase in MMSE†	
	N, %	11 241 (56.99)		6252 (31.69)	
		OR (95% CI)	P value	OR (95% CI)	P value
Positive change in NDVI	9997 (50.68)	Reference	/	Reference	/
Negative change in NDVI	9729 (49.32)	1.25 (1.18 to 1.34)	<0.001	0.90 (0.84 to 0.96)	<0.001

A decrease in MMSE was defined as lower MMSE score at the final survey than at the baseline, while an increase in MMSE indicated higher MMSE score at the final survey. Additionally, all the regression models were adjusted for age, gender, ethnicity, marital status, urban/rural residence, education, occupation, financial support, social and leisure activity, smoking status, alcohol consumption, physical activity and MMSE score at baseline.

*Reference group: Participants without changes in MMSE or had an increase in MMSE during the follow-up period.

†Reference group: Participants without changes in MMSE or had a decrease in MMSE during the follow-up period.

MMSE, Mini-Mental State Examination; NDVI, Normalised Difference Vegetation Index.

## Discussion

In this study of 38 327 older adults aged 65 years and older in China, we observed a protective association between residential greenness and cognitive function. We also found that participants living in areas with decreasing greenness were more likely to experience decreases in MMSE scores. The effect size of each 0.1-unit increase in NDVI was roughly equivalent to 1 year of ageing, as the 1-year increase in age was associated with 0.28-point decrease in MMSE score, and an OR of 1.07 of cognitive impairment. If greenness can indeed delay cognitive decline in older adults, these findings have important policy implications for preventing dementia and AD in China, as the green space can become a tool for prevention and mitigating cognitive impairment among older adults.

There are only a few studies on residential greenness and cognitive function, which is likely due to the difficulties in assembling large cohorts with individual cognitive measurements. Past studies have found varying results on the association between access to greenness and cognitive function.^9^ The Lothian Birth Cohort 1936 composed of 1091 adults aged 70 years and older in Scotland reported a higher percentage of parks in the 1500 m radius around their residences in childhood and adulthood was related to better cognitive ageing.^9^ A study of 884 adults aged 65 years and older in the USA showed that the participants who walked in the parks had significantly higher MMSE scores than those walking in the shopping malls and indoor gyms.^8^ However, other cross-sectional studies showed conflicting evidence. In Chicago, there is no association between more park areas and cognitive function among 949 adults aged 50 years and older, assessed by the Telephone Instrument for Cognitive Status.^10^ A study of 6658 adults aged 40–69 years in Quebec, Canada, also reported no association between NDVI values in the 500 m radius around the residence, and cognitive function evaluated through the reaction time, working memory and executive function.^11^ A study in the UK showed that a higher percentage of green space was associated with higher odds of cognitive impairment (defined as MMSE score<25) in 2424 participants aged 65 years and older. The authors pointed out that older adults living in the community with large green space might be isolated and have lower cognitive stimulation, or that older adults with cognitive impairment were likely to stay in the community with more green space.^12^

Our study showed that residential greenness had a small protective effect on cognitive function among older adults. The unique data structure of the CLHLS allowed us to use a range of analytical techniques, including the linear regression and the logistic regression. Second, since prior studies did not or could not assess changes in area-level greenness, our additional piece of evidence on how changes in NDVI affect MMSE reinforces the cross-sectional analysis. China is unique in the sense that there is a rapid expansion of built-up areas due to the economic development, and thus, we are able to see changes in area-level greenness. Lastly, we also used a longitudinal data analysis approach of the mixed effects logistical regression, but it did not find a statistical significance, perhaps due to a higher mortality rate of those with lower MMSE scores over time, or perhaps this method has better or worse model specifications compared with the other analysis methods.

A secondary endpoint of our analysis is to determine whether there might be differential subgroup effect of residential greenness on cognitive function. We looked at possible effect modification by SES, gender and physical activity. We did not find the effect modification by SES (p values for interaction between NDVI and urban or rural residence, occupation, education and financial support were 0.168, 0.203, 0.554 and 0.597, respectively). The participants with the lowest SES had the strongest effect in the Lothian Birth Cohort in Scotland.^9^ Two other studies in the UK also showed a lower inequality in all-cause mortality and psychological health among participants exposed to higher levels of greenness.^26 27^ The potential mechanisms could be that higher SES populations have many venues for health-seeking behaviour, whereas utilisation of green space is of greater contribution to the health in lower SES groups.^27^ Second, the effect modification of gender is unclear in our study because our linear regression and logistic regression show inconsistent findings. The inconsistency may be due to different model specifications of the linear regression and the logistic regression. In our study, protective effects of residential greenness on cognitive function were similar between males and females, although males had higher MMSE scores than females (24 vs 19) and their residential greenness was similar at baseline. It is likely that males and females have different perception and usage of green space. Whether there is a gender difference in the relationship between greenness and cognitive function is also unclear in other studies. Gender difference remains to be understood. Third, we also found a stronger association between residential greenness and cognitive function among older adults who did not exercise (OR 0.93, 95% CI 0.90 to 0.95) compared with those who did exercise (OR 0.97, 95% CI 0.94 to 1.01). This is most surprising to us because our prior belief is that those who exercise can benefit more from green space. Furthermore, evidence shows that living in greener areas promotes more physical activity.^4 28^ But, this relationship may not extend across all populations as another study of 4899 participants in the Netherlands pointed out that residential greenness was barely associated with physical activity.^29^ Additionally, some studies argued that the association between green space and health probably was more likely to be explained by stress reduction and social cohesion, instead of physical activity.^18 29 30^ It is puzzling to us that those who did not exercise, experienced more benefits from residential greenness.

While we believe our study has the most informative exposure and outcome measurements. It has some limitations that affect our interpretation. First, NDVI values are general area-level greenness and cannot reflect the different types of vegetation or space for activity. We do not know activity patterns of participants, and whether or not different types of vegetative have different effects on cognitive function. We did not examine the association between residential greenness and cognitive function in other scales, and do not know whether different scales have stronger or weaker association. Second, although the MMSE is a quantitative assessment of cognitive impairment, it’s scoring system may be insensitive to mild cognitive impairment. The different versions, administration, scoring and interpretation could also cause inconsistencies,^24^ although the adapted Chinese version used in our study was demonstrated as reliable and valid.^15^ Third, there may be an issue with informative censoring in the analysis of changes in NDVI and in MMSE since only the participants with follow-up surveys were included. Compared with the participants without any follow-up survey, mostly due to mortality events, those with follow-up surveys were younger and had better cognitive function at baseline (see online supplementary table 1). However, we do not think this biases our results away from the null effect. Fourth, we did not study the effects of diet on the association, although evidence shows that diet may influence cognitive function. For instance, studies reported that the Mediterranean diet was related to slower cognitive decline, and better cognitive function. However, evidence based on the observational studies is not consistent, while evidence reported by randomised controlled trials is limited.^31^ Additionally, diet is unlikely to mediate the association between residential greenness and cognitive function in our study.

There are several strengths to our study. To the best of our knowledge, our study is the first study in China to examine the effects of residential greenness on cognitive function. We assessed residential greenness by calculating NDVI values based on participants’ residential addresses. In some prior studies, greenness exposure was measured by proximities to natural and green space. The use of aerial satellite-derived measurements allowed us to track changes in greenness over time, track many individuals in a large cohort and be able to measure greenness objectively. Our study population is highly immobile and less likely to be affected by relocation because of their advanced age and social benefits connected to the household registration system.^32 33^ Additionally, we had a large sample as 38 327 participants were recruited from 22 provinces in the mainland, China. Our study also had a balanced age structure. A total of 22.25%, 26.51%, 27.18% and 24.06% were young–old, octogenarians, nonagenarians and centenarians, respectively. This age distribution could help to understand the association among different age groups of older adults. Furthermore, our study is unique compared with the studies in developed countries because the participants with lower SES live in greener areas in China. Since higher SES like higher education was closely related to better cognitive function, SES was less likely to bias the association between residential greenness and cognition.

## Conclusion

We found that a higher level of residential greenness was related to better cognitive function, and also slower cognitive decline among Chinese older adults. Each 0.1-unit increase in NDVI is roughly equivalent to 1 year of ageing with respect to cognitive decline. Involving more green space in city planning during the process of urbanisation may reduce cognitive decline and subsequently, cases of AD and dementia, which would, in turn, reduce healthcare expenditures in China.
