# Supplementary material for: Association between residential greenness and cognitive function: analysis of the Chinese Longitudinal Healthy Longevity Survey
Source: BMJ Nutr Prev Health. 2019 Aug 26;2(2):72–9. doi: 10.1136/bmjnph-2019-000030 (PMC7664503; doi:10.1136/bmjnph-2019-000030)
Supplement: Supplementary data [file bmjnph-2019-000030supp001.pdf]

Supplementary Table 1. Baseline characteristics of participants with follow-up, and without follow-up survey

Characteristics	Participants with follow-up	Participants without follow-up	p value
N	19,726	18,601	
Baseline MMSE (mean±SD)	23.45±7.89	17.71±10.51	<0.0001
Baseline NDVI (mean±SD)	0.40±0.15	0.39±0.15	<0.0001
Age (years) (mean±SD)	84.28±11.31	92.17±10.12	<0.0001
Age group (n, %)			
65-79	6,366 (32.27)	2,162 (11.62)	
80-89	6,282 (31.85)	3,879 (20.85)	<0.0001
90-99	4,434 (22.48)	5,984 (32.17)	
>=100	2,644 (13.40)	6,576 (35.35)	
Gender (n, %)			
Males	8,714 (44.18)	7,033 (37.81)	<0.0001
Females	11,012 (55.82)	11,568 (62.19)	
Ethnicity (n, %)			
Han	18,506 (93.82)	17,650 (94.89)	<0.0001
Others	1,220 (6.18)	951 (5.11)	
Marital status (n, %)			
Married and living with spouses	7,036 (35.67)	3,251 (17.48)	<0.0001
Not married	12,690 (64.33)	15,350 (82.52)	
Residence (n, %)			
Urban area	4,270 (21.65)	4,806 (25.84)	<0.0001
Rural area	15,456 (78.35)	13,795 (74.16)	
Occupation (n, %)			
Professional work	1,586 (8.04)	1,073 (5.77)	<0.0001
Non-professional work	18,140 (91.96)	17,528 (94.23)	
Education (n, %)			
Formal education	7,847 (39.78)	5,500 (29.57)	<0.0001
No formal education	11,879 (60.22)	13,101 (70.43)	
Financial support (n, %)			
Financial independence	5,792 (29.36)	3,350 (18.01)	<0.0001
Financial dependence	13,934 (70.64)	15,251 (81.99)	
Social and leisure activity index (mean±SD)	2.45±1.50	1.67±1.50	<0.0001

<0.0001

Smoking status (n, %)			
Yes	4,038 (20.47)	2,766 (14.87)	
No	15,688 (79.53)	15,835 (85.13)	
Alcohol consumption (n, %)			
Yes	4,266 (21.63)	3,117 (16.76)	<0.0001
No	15,460 (78.37)	15,484 (83.24)	
Physical activity (n, %)			
Yes	6,537 (33.14)	4,386 (23.58)	<0.0001
No	13,189 (66.86)	14,215 (76.42)	
Geographic region (n, %)			
Central China	2,877 (14.58)	2,703 (14.53)	
Eastern China	7,861 (39.85)	7,630 (41.02)	
Northeastern China	1,403 (7.11)	1,519 (8.17)	<0.0001
Northern China	926 (4.69)	996 (5.35)	
Northwestern China	254 (1.29)	258 (1.39)	
Southern China	3,853 (19.53)	3,078 (16.55)	
Southwestern China	2,552 (12.94)	2,417 (12.99)	

Supplementary Table 2: Survey-weighted regression on(Weighted) Association between residential

	Serial cross-sectional analysis		Longitudinal analysis	
Exposure metric	Linear regression	Logistic regression	Linear mixed-effects regression	Logistic mixed-effects regression
	Coefficient for change in MMSE score (95% CI)	OR of cognitive impairment (95% CI)	Coefficient for change in MMSE score (95% CI)	OR of cognitive impairment (95% CI)
0.1*NDVI	0.18 (0.11, 0.25)	0.93 (0.90, 0.97)	0.060 (-0.00075, 0.12)	0.98 (0.94, 1.01)
Quartiles of NDVI				
Quartile 1	Reference	Reference	Reference	Reference
Quartile 2	0.22 (-0.033, 0.47)	0.93 (0.80, 1.08)	-0.0022 (-0.23, 0.22)	1.03 (0.90, 1.18)
Quartile 3	0.24 (-0.036, 0.53)	0.95 (0.81, 1.11)	-0.070 (-0.31, 0.17)	1.06 (0.92, 1.22)
Quartile 4	0.81 (0.52, 1.09)	0.71 (0.60, 0.84)	0.30 (0.057, 0.55)	0.87 (0.75, 1.01)

Note: All the regression models were adjusted for age, gender, ethnicity, marital status, urban/rural residence, education, occupation, financial support, social and leisure activity, smoking status, alcohol consumption, and physical activity at baseline. The longitudinal analysis was additionally adjusted for the time to reflect the number of years for each follow-up survey since entering the cohort.
